# Quaternary ammonium disinfectants and antiseptics: tolerance, resistance and potential impact on antibiotic resistance

**DOI:** 10.1186/s13756-023-01241-z

**Published:** 2023-04-13

**Authors:** John M. Boyce

**Affiliations:** J.M. Boyce Consulting, LLC, 5123 Town Place, Middletown, CT Connecticut, USA

**Keywords:** Quaternary ammonium compounds, Disinfectants, Antiseptics, Side effects, Tolerance, Resistance, Antibiotic resistance

## Abstract

**Background:**

Due to the substantial increase in the use of disinfectants containing quaternary ammonion compounds (QACs) in healthcare and community settings during the COVID-19 pandemic, there is increased concern that heavy use might cause bacteria to develop resistance to QACs or contribute to antibiotic resistance. The purpose of this review is to briefly discuss the mechanisms of QAC tolerance and resistance, laboratory-based evidence of tolerance and resistance, their occurrence in healthcare and other real-world settings, and the possible impact of QAC use on antibiotic resistance.

**Methods:**

A literature search was conducted using the PubMed database. The search was limited to English language articles dealing with tolerance or resistance to QACs present in disinfectants or antiseptics, and potential impact on antibiotic resistance. The review covered the period from 2000 to mid-Jan 2023.

**Results:**

Mechanisms of QAC tolerance or resistance include innate bacterial cell wall structure, changes in cell membrane structure and function, efflux pumps, biofilm formation, and QAC degradation. In vitro studies have helped elucidate how bacteria can develop tolerance or resistance to QACs and antibiotics. While relatively uncommon, multiple episodes of contaminated in-use disinfectants and antiseptics, which are often due to inappropriate use of products, have caused outbreaks of healthcare-associated infections. Several studies have identified a correlation between benzalkonium chloride (BAC) tolerance and clinically-defined antibiotic resistance. The occurrence of mobile genetic determinants carrying multiple genes that encode for QAC or antibiotic tolerance raises the concern that widespread QAC use might facilitate the emergence of antibiotic resistance. Despite some evidence from laboratory-based studies, there is insufficient evidence in real-world settings to conclude that frequent use of QAC disinfectants and antiseptics has promoted widespread emergence of antibiotic resistance.

**Conclusions:**

Laboratory studies have identified multiple mechanisms by which bacteria can develop tolerance or resistance to QACs and antibiotics. *De novo* development of tolerance or resistance in real-world settings is uncommon. Increased attention to proper use of disinfectants is needed to prevent contamination of QAC disinfectants. Additional research is needed to answer many questions and concerns related to use of QAC disinfectants and their potential impact on antibiotic resistance.

## Introduction

Quaternary ammonium compounds (QACs) intended for preservative and antimicrobial applications are present in a wide variety of products, resulting in potential exposures in many settings [1, 2]. For example, QACs can be found in personal care products, ophthalmic medications, skin antiseptics, and disinfectants used in homes and healthcare facilities [1, 2]. QACs comprise a large number of chemical structures, with many variations on the basic structure, which includes a nitrogen (head) attached with four bonds to alkyl or aryl chains (tails) with varying number of carbon atoms. Examples of several types of commonly used QACs are listed in Table 1.

**Table 1:** Examples of quaternary ammonium compounds commonly used in disinfectants

Compound	Abbreviations	CAS Number
Akyl dimethyl benzyl ammonium chloride frequently called benzalkonium chloride (BAC)	ADBAC C10-C18, or BAC C10-C18	8001-54-5
Didecyl dimethyl ammonium chloride Dioctyl dimethyl ammonium chloride	DDAC C10 DDAC C8	7173-51-5 3026-69-5
• Quaternary ammonium compounds, C_12 − 18_-alkyl[(ethylphenyl)methyl]dimethyl, chlorides	ADEBAC C10-C18	68956-79-6
Alkyl trimethyl ammonium chloride	ATMAC C12-C18	68391-03-7
Cetyl trimethyl ammonium chloride	CTAC	112-02-7
Cetylpyridinium chloride	CPC	123-03-5
Benzethonium chloride	BZT	121-54-0

**Fig. 1:**
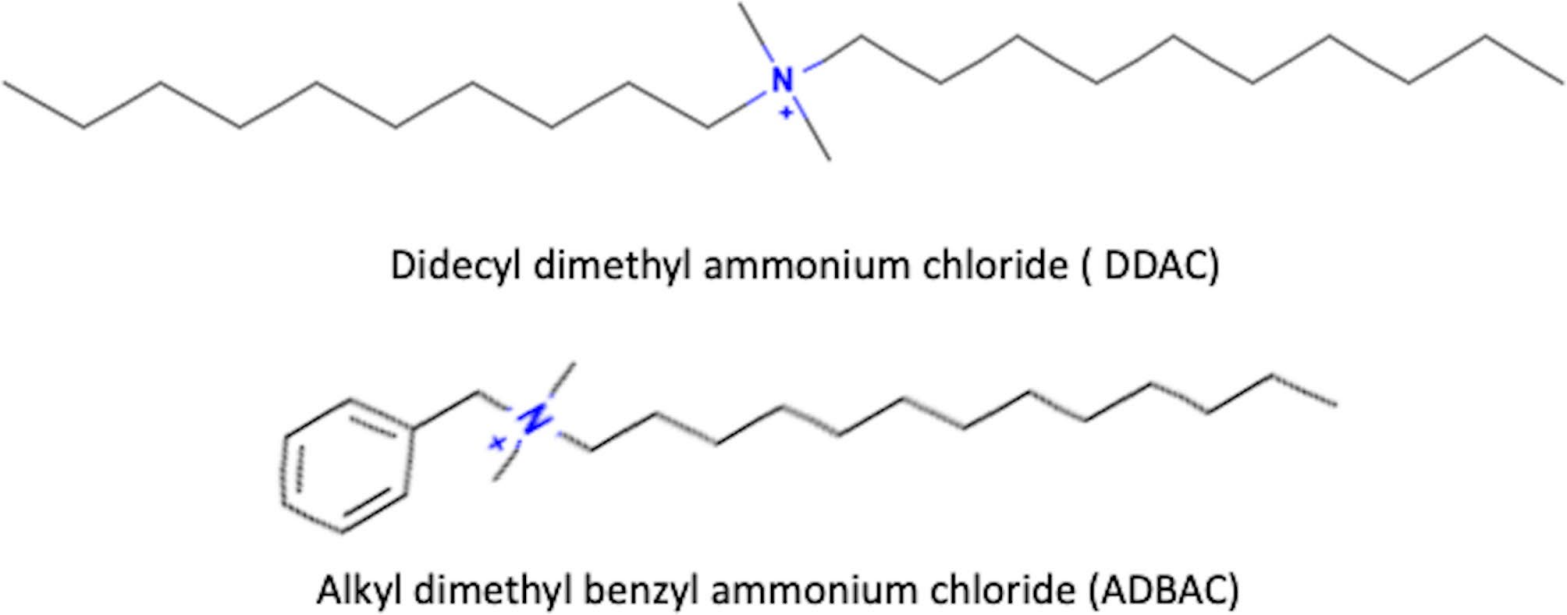
Structure of (**A**) didecyl dimethyl ammonium chloride (DDAC) and (**B**) alkyl dimethyl benzyl ammonium chloride (ADBAC).

The chemical structures of two QACs commonly used in disinfectants (didecyl dimethyl ammonium chloride (DDAC) and alkyl dimethyl benzyl ammonium chloride (ADBAC or BAC) are shown in the Fig. 1.

More complicated structures such as bis- and tris-QACs with two or three nitrogen atoms or QACs attached to polymers (polymeric QACs) are beyond the scope of this review.

Recently, interest in these compounds has intensified due to the large number of QAC disinfectants recommended for use against SARS-CoV-2 and their widespread use during the COVID-19 pandemic [2–4]. For example, QAC-containing disinfectants comprised 277/597 (46.3%) of the United States Environmental Protection Agency (EPA) List N products listed as effective against SARS-CoV-2 [5]. Concerns include misuse of disinfectants, a 331% increase in QAC levels of untreated wastewater in one area, QAC levels in household dust that were significantly higher than levels found before the pandemic, and the finding of QACs in blood and breast milk samples collected from individuals during the pandemic [3, 5–9]. Furthermore, marketing and ubiquitous use in community settings (e.g., households, offices) and healthcare facilities of QACs, including products with persistent activity due to silane moieties, during the pandemic might result in even higher or more prolonged sub-inhibitory concentrations on surfaces and in the environment, which may be a risk factor for emergence of QAC tolerance or resistance, and perhaps to an increase in antibiotic tolerance or resistance among pathogenic bacteria [1, 4, 10, 11]. One QAC-silane disinfectant was taken off the market because the manufacturer was selling and distributing the product in ways that were inconsistent with federal regulations [12]. This review focuses on mechanisms of tolerance and resistance of bacteria to QACs, and addresses concerns about the possible influence of QAC use on antibiotic resistance. Emphasis is placed on QAC compounds that are commonly used in disinfectants and antiseptics used in healthcare and community settings.

## Methods

A literature search of the PubMed database was performed using the following search terms: quaternary ammonium compounds + disinfectants + tolerance; quaternary ammonium compounds + disinfectants + resistance; quaternary ammonium compounds + antiseptics + tolerance; quaternary ammonium compounds + antiseptic + resistance; and quaternary ammonium compounds + contaminated disinfectants. The search was limited to English language citations, and the search was confined to the period 2000 to mid-January 2023. A total of 1,161 unique citations were retreived from PubMed. Following a review of titles, 386 abstracts were reviewed, and 328 full-text articles were downloaded and reviewed. Additional citations were identified in bibliographies of reviewed articles. Citations dealing with issues related to the potential impact on the environmental pollution were not included. Data from 138 articles are included in the present review.

## Results

Unfortunately, the terms such as QAC tolerance, reduced susceptibility, and resistance are not always used consistently by the scientific community, and can sometimes be misleading. As pointed out by Maillard, [13] the term “resistance” has often been defined as an increase in the in vitro minimum inhibitory concentration (MIC) or minimum bactericidal concentrations (MBC) of a disinfectant or an antiseptic. However, despite relatively small increases in the MIC (or MBC) of the QAC ingredient, products may still be very effective because the in-use concentration is often significantly higher than the in vitro MIC or MBC. For example, some Gram-negative bacteria have MICs to BAC of 12 to 60 µg per mL, [14–16] while QAC-based disinfectants may have in-use concentrations ranging from 200 to 16,000 ug/ml) [3, 17]. Accordingly, bacteria manifesting several-fold increase in the MIC of a disinfectant are more appropriately referred to as being tolerant or having reduced susceptibility to the disinfectant. For the purposes of this review, the term tolerance will be used to denote reduced susceptibility, and a biocide-resistant microorganism is defined as one that is not inactivated by an in-use concentration of a biocide [13, 18]. For more in-depth discussions of QAC tolerance and resistance and possible impact on antibiotic resistance, several reviews are available [1, 13, 19, 20].

### Methods for studying QAC tolerance and resistance

A variety of methods have been used to study tolerance and resistance to QAC disinfectants and antiseptics [13, 21]. A few studies have evaluated bacteria recovered from surfaces which have been disinfected with QAC-based disinfectants [22, 23]. Two studies have evaluated the mechanisms of resistance in QAC-resistant strains recovered from contaminated QAC disinfectants or antiseptics [24, 25]. A much more common method is to study the adaptive changes that occur when bacteria are exposed in vitro to increasing, sublethal concentrations of QACs [15, 16, 26–30]. Although the extent to which such in vitro experiments yield the same findings that occur in nature has been questioned, considerable insight into the mechanisms of biocide resistance has been gained by studying such adaptive changes [24, 31].

### Mechanisms of QAC tolerance and resistance

#### Intrinsic tolerance and resistance

Spore-forming organisms and mycobacteria possess intrinsic resistance to disinfectants and antiseptics due to their complex cell wall structures [32]. Gram-negative bacteria are generally less susceptible than Gram-positive bacteria to QACs due to their outer membrane, which can make it somewhat more difficult for biocides to reach their target site (cytoplasmic membrane) [32, 33]. In general, QACs are classified as fungicidal and viricidal against enveloped viruses, but have poor activity against mycobacteria and non-enveloped viruses such as norovirus [34, 35]. However, some QAC-based products are less active than some other disinfectants against *Candida* species including *C. auris.* [36] Intrinsic biocide resistance can also be due to the presence of chromosomally-encoded efflux pumps, the ability of organisms to produce biofilm, or degradation of QAC compounds [37, 38]. Examples of bacteria that often have intrinsic reduced susceptibility to QACs include species of *Pseudomonas* and *Burkholderia*, [32, 38–40] which may explain in part the frequency with which they have been reported to cause contamination of QAC-based antiseptic and disinfectant solutions [41]. The frequency and mechanisms of decreased susceptibility or resistance to QACs among fungi and viruses are outside the scope of this review.

#### Membrane changes

In experiments involving exposure of a *Pseudomonas aeruginosa* strain to increasing concentrations of BAC-C14 or didecyldimethylammonium bromide (DDAB), changes in the cytoplasmic membrane fatty acid composition were associated with increases in the MIC and MBC to both compounds, which subsequently decreased when the organism was grown in QAC-free media [14, 42]. A *Listeria monocytogenes* strain exposed to BAC developed changes in cell surface fatty acid composition, which may have contributed to a two-fold increase in BAC MIC [43]. BAC can cause increases in membrane fluidity among some strains of bacteria recovered from food sources [44]. A study involving proteomic assays found that exposure to increasing concentrations of BAC resulted in changes in proteins that have been associated with acquired antimicrobial resistance, and decreased expression of porins and lipoproteins [45]. Strains of *Pseudomonas* species with reduced susceptibility to QACs have been reported to have changes in their outer membrane proteins and lipopolysaccharide [46, 47]. A 2018 study by Kim et al. [27] used genomic and transcriptomic methods to assess the impact of exposing *Pseudomonas aeruginosa* to increasing concentrations of BAC found that the strain was able to survive concentrations of BAC up to 1200 to 1600 ppm (ug/ml). Adaptation resulted in decreased expression of porins related to BAC transport, reduced growth rate, and reduction in the membrane negative charge. Similarly, analysis of a strain of *Pseudomonas fluorescens* recovered from a contaminated BAC solution revealed a reduction in adsorption of BAC to the cell surface due to a decrease negative cell surface charge [24]. Growth of *Salmonella enterica* serovar *enteritidis* in BAC resulted in decreased susceptibility to the agent, alterations in cell surface roughness and a shift in fatty acid composition [48]. Exposure of *E. coli* to increasing concentrations of BAC has been shown to produce a subpopulation of surviving cells with a decreased growth rate and *lpxM* gene mutation-related decrease in negative surface charge that likely decreased adsorption of BAC to its target site [31]. The authors concluded that their findings suggest that episodes of incomplete disinfection that result in a subpopulation of surviving cells could adversely affect the efficacy of disinfection practices.

#### Efflux pumps

Many bacteria contain efflux pumps that can remove substances such as QACs and various antibiotics from the cytoplasm and cytoplasmic membrane [37, 49]. There are several classes of efflux pumps, including resistance-nodulation-division (RND) superfamily, major facilitator superfamily (MFS), ATP-binding cassette (ABC) family, small multidrug resistance (SMR) family, multidrug and toxic compound extrusion (MATE) family, and proteobacterial antimicrobial compound efflux (PACE) family [50, 51]. RND efflux pumps, which are most commonly found in Gram-negative bacteria, are composed of a cytoplasmic membrane pump, a periplasmic protein, and an outer membrane protein channel [49]. For example, some clinical *E. coli* strains have been shown to contain a class 1 integron with cassettes containing genes encoding for trimethoprim-sulfamethoxazole resistance (*drfA1/sul1)* and the qacE∆1gene that encodes for QAC tolerance [52]. Reduced susceptibilities were due to overexpression of AcrAB-TolC efflux pump regulatory genes *tolC* and *marOR*, which was also associated with decreased susceptibility to ciprofloxacin. The MATE family pump (PmPM) can be found in *P. aeruginosa, Acinetobacter baumannii*, and *Vibrio parahaemolyticus* as well as *in S. aureus.* [50] MFS, ABC and SMR family efflux pumps are the most common in Gram-positive bacteria, although a MFS pump (SmfY) has also been found in *Serratia marcescens* [53]. In staphylococci, six different plasmid-mediated efflux pumps (QacA, QacB, QacC, QacG, QacH and QacJ) can reduce susceptibility to QACs, but do not result in QAC resistance [54]. The qacH gene has also been identified on a non-classic class 1 integron located on a conjugative plasmid in *Proteus mirabilis* [55]. The gene *qacH* and *bcrABC* cassette, which both encode for QAC efflux, have been found on transposable elements in different strains of *Listeria monocytogenes.* [56, 57] Some examples of the efflux pumps found in common pathogenic bacteria are listed in Table 2 [15, 49–51, 54, 58–65].

**Table 2:** Examples of efflux pumps conferring increased tolerance to quaternary ammonium compounds. *Cite at line 238*

Pathogen	RND	MFS	ABC	SMR	MATE	PACE
E. coli	AcrAB-TolC	MdfA(Cmr), EmrB, EmrD		EmrE, SugE	NorM, MdtK/YdhE	
Pseudomonas	Mex-Opr			QacE△1	PmPM	
Serratia	SdeXY, SdeAB, SdeIJ,	SmfY				
Burkholderia	Mex-Opr-like					AceI
Acinetobacter		QacA, QacB		QacE, QacE△1	PmPM	AceI
Achromobacter						
Proteus					PmPM	
Vibrio					PmPM	
Aeromonas				QacE2		
Enterobacter	acrB			SugE	EmmdR/YeeO	
Klebsiella				QacE		
Salmonella typhimurium	AcrAB-TolC					
		MFS	ABC	SMR		
S. aureus		NorA, QacA, Qac B; MepA MdeA	EfrAB	QacC,QacG,QacH, QacJ, QacE△1		
L. monocytogenes				EmrE_Lm_		
Enterococcus		QacA/B	EfrAB	QacC, QacE△1		

RND = resistance-nodulation-division; MFS = major facilitator superfamily; ABC = ATP-binding cassette; SMR = small multidrug resistance; PACE = proteobacterial antimicrobial compound efflux; MATE = multidrug and toxic compound extrusion

It is worth noting that decreased susceptibility, and occasionally resistance, to QACs is likely mediated by efflux pumps in addition to other mechanisms. For example, a number of studies suggest that efflux pumps may promote, directly or indirectly, biofilm production [66, 67].

#### Biofilm and reduced susceptibility to QACs

The presence of biofilm reduces the susceptibility to disinfectants in both Gram-positive and Gram-negative bacteria, with the greatest impact in Gram-negative bacteria [26, 68–71]. Reduced susceptibility to biocides in biofilms may be due to several factors [71]. Multiple layers of bacteria and the extracellular matrix may result in conditions in which effective biocide concentrations do not reach all internal layers of cells [72]. Diffusion of positively charged disinfectants within negatively charged biofilms could be hindered because of electrostatic interactions [73]. Biofilms may promote induction or upregulation of efflux pumps and increased mutations that may affect biocide susceptibility [66, 71, 74]. Decreased bacterial growth rates in biofilm may also result in reduced susceptibility to disinfectants [72]. As a result, bacteria present in biofilms may have minimum bactericidal concentrations 10-1000 times higher than planktonic organisms of the same strains, and in some instances may not be killed by biocides in concentrations recommended by the manufacturer [68]. Accordingly, it is important clean surfaces (i.e., remove organic and inorganic material) as part of the disinfection process [75].

#### Degradation of QACs

Studies of multiple strains of *Burkholderia* species have documented that these organisms can partially degrade BAC over a period of 7 days [38]. Multiple constitutively expressed enzymes were involved in degradation of BAC. In a few organisms such as *Pseudomonas nitroreducens* and *Pseudomonas fluorescens*, biocide resistance may also be due in part to enzymatic degradation of QACs [13, 38, 76, 77].

### Adaptive studies

Multiple studies have demonstrated that exposure of bacteria to subinhibitory concentrations of QAC resulted in increased QAC MICs or MBCs, which has often been due to upregulation of QAC efflux pumps [15, 16, 26, 27, 29, 49, 78]. In a number of such studies, MICs or MBCs have increased to levels that are often far below in-use concentrations, with variable degrees of stability of the increased MICs when the organism is no longer exposed to the QAC [14, 15, 26, 29, 31, 43, 44, 69, 79]. However, some experts have argued that even temporary (a few days or weeks) increases in MICs to levels lower than in-use concentrations of disinfectants might affect the efficacy of standard surface disinfection protocols [29]. In contrast, a few adaptive studies found that some strains developed stable BAC MICs that are in the range of in-use concentrations [27, 30, 80]. For example, in one adaptive study in which *Pseudomonas aeruginosa* strains were exposed to increasing concentrations of BAC, several strains were able to survive concentrations from 1240 to 1640 µg/ml, which are concentrations higher than the recommended in-use concentrations of some QAC-based disinfectant products [3, 25, 27]. Although the MICs of a majority of strains reverted back to baseline levels within 150 generations or less when grown in the absence of BAC, several strains maintained high MICs. Additional studies, including transcriptome sequencing analysis, of a *Pseudomonas aeruginosa* strain exposed for 3 years to subinhibitory BAC concentrations and a similar unexposed strain revealed changes that included reduced growth rate, decreased expression of porins associated with BAC transport, and upregulation of efflux genes [81]. Mutations in the *pmrB* gene, which in other studies has been shown to result in reduction in the negative charge of the outer membrane, also occurred. Adaptive studies have also found that exposure of bacteria to QACs can promote biofilm formation [26].

A few adaptive studies have resulted in antibiotic tolerance or resistance [5, 78, 82–84].

Continuous exposure of *Pseudomonas* aeruginosa to BAC resulted in an increase in BAC MIC to 350 ug/ml and of ciprofloxacin to 32 ug/ml [78]. The latter change was attributed to a mutation in the *nfxB* gene, which is an efflux pump regulator. In another study, step-wise exposure of *E. coli* to DDAC resulted in tolerance to DDAC and BAC, and development of clinically-defined resistance to chloramphenicol, ampicillin, ciprofloxacin, cefotaxime and ceftazidime [84]. In some strains, an increase expression of *acrB* gene likely contributed to emergence of antibiotic resistance. In studies by Jia et al., [5] exposure to DDAC was more likely than BAC to result in resistance to some antibiotics in a laboratory strain of *E. coli*, albeit some of the resistance was to antibiotics seldom used to treat *E. coli* infections. Whole genome sequencing revealed that mutations affecting RNA transcription, efflux pump regulators, and cell membrane synthesis had occurred [5]. Rakic-Martinez et al. [82] found that exposure of *Listeria monocytogenes* to BAC yielded strains that developed efflux pump-mediated resistance to gentamicin, which is used for treatment of invasive *Listeria* infections. Another study of *L. monocytogenes* found that exposure to BAC or DDAC caused increased tolerance to ciprofloxacin MICs, [85] which was later shown to be caused by mutations in the *fepR* repressor that affects the fluoroquinolone efflux pump FepA [86]. It should be noted that the stability of antibiotic resistance demonstrated in the above studies was tested over short time periods in some studies, but none evaluated the persistence of resistance over prolonged time periods.

A proposed protocol that exposes pathogens to in-use concentrations of products has been developed to better predict bacterial resistance to biocides [87]. The protocol recommends that if an organism develops a stable increase in MIC or MBC, several avenues of investigation should be performed since biocide resistance is usually due to several mechanisms. An alternative approach that warrants further attention is to study the impact of QAC exposure on the adaptive changes in biocide susceptibility by exposing multi-organism biofilms (such as those found in sink drains) to in-use concentrations of formulated disinfectant products, rather than exposure to pure QAC compounds [88].

### QAC tolerance in real-world settings

Some authors have mentioned that it is arguable whether biocides can develop tolerance in non-laboratory settings [69]. However, there are a few examples of QAC tolerance in real-world settings. One study found that hospital isolates of *Pseudomonas* aeruginosa had increased BAC tolerance, and some of the isolates also had clinically-significant resistance to antibiotics [89]. He et al. [22] reported that 63 isolates of *Staphylococcus* spp. recovered from fitness centers and school dormitories had slightly increased tolerance to BAC, which was attributed to the presence of one or more *qac* genes. A study of 199 *A. baumannii* clinical isolates from four teaching hospitals found that some isolates had BAC MICs and MBCs as high as 640 to 1280 ug/ml, respectively [90].

### QAC resistance in real-world settings: contaminated in-use disinfectants and antiseptics

QAC-resistant microorganisms have been defined as those which are capable of surviving in an in-use concentration of a product [13]. If one adopts this definition, then examples of QAC-resistant bacteria include those recovered from contaminated in-use disinfectants or antiseptic solutions in clinical settings [25, 41, 91–115]. Product contamination by QAC-resistant pathogens can also be affected by user errors, including use of outdated products, substantial over-dilution of concentrated solutions, or introduction of organic material or prolonged soaking of wipes with strong QAC-binding affinity in disinfectant solutions [13, 41, 116, 117]. Of 43 reported episodes of contaminated disinfectants or antiseptics, authors described outbreaks of infection (26), single cases of infection (3), pseudo-outbreaks (4) surveys of disinfection buckets (3), contamination without reported confirmed consequences (6), and an episode with unclear outcome (1) (see Table 3).

**Table 3:** Reported episodes of contaminated quaternary ammonium disinfectant or antiseptic. *Cite at line 341*

Year	Author	Pathogen	Product Type	Type of Report
1951	Lowbury	*Pseudomonas pyocyanea*	Antiseptic	Infection outbreak
1957	Keown	*Pseudomonas aeruginosa*	Disinfectant	Infection outbreak
1958	Plotkin	*Pseudomonas*	Antiseptic	Infection outbreak
1959	Shickman	*Pseudomonas aeruginosa*	Disinfectant	Single infection
1960	Malizia	*Enterobacter aerogenes*	Antiseptic	Infection outbreak
1961	Lee	*Pseudomonas/ Achromobacter group*	Antiseptic	Infection outbreak
1967	Burdon	*Pseudomonas multivorans*	Antiseptic	No confirmed infections
1969	CDC*	*Pseudomonas kingii*	Antiseptic	Infection outbreak
1970	Hardy	*Pseudomonas EO-1*	Disinfectant	Infection outbreak
1970	Bassett	Burkholderia cepacia **	Antiseptic	Infection outbreak
1970	Gilardi	*Pseudomonas EO-1*	Antiseptic	Outcome unclear
1976	Dixon	*Pseudomonas* species	Disinfectant	Infection outbreak
1976	Kaslow	*Burkholderia cepacia; Enterobacter*	Antiseptic	Pseudo-outbreak
1976	Frank	*Burkholderia cepacia*	Antiseptic	Infection outbreak
1976	Morris	*Burkholderia cepacia*	Antiseptic	Single infection
1976	Guinness	*Burkholderia cepacia*	Antiseptic	Infection outbreak
1976	Wishart	*Stenotrophomonas maltophilia*	Antiseptic	Infection outbreak
1980	Ehrenkranz	*Serratia marcescens*	Disinfectant	Infection outbreak; contaminated surfaces
1981	Fox	*Serratia marcescens*	Antiseptic	Infection outbreak (dogs and cats)
1982	Van Damme	*Serratia marcescens*	Antiseptic	Outbreak of bovine mastitis infections
1984	Sautter	*Serratia marcescens*	Antiseptic	Single infection
1987	Nakashima	*Serratia marcescens*	Antiseptic	Infection outbreak
1988	Gahrn-Hansen	*Achromobacter xylosoxidans*	Disinfectant	Infection outbreak
1990	Georgia DPH	*Mycobacterium chelonae*	Antiseptic	Infection outbreak
1996	Nagai	*Pseudomonas fluorescens*	Disinfectant	No infections
1996	Oie	*Burkholderia cepacia; Pseudomonas aeruginosa; Pseudomonas fluorescens*	Antiseptic and Disinfectant	No Infections
1999	Olson	*Pseudomonas aeruginosa*	Disinfectant	Infection outbreak
2000	Kaitwatcharachai	*Burkholderia cepacia*	Disinfectant	Infection outbreak
2002	Lehours	*Achromobacter xylosoxidans*	Disinfectant	Infection outbreak
2003	Tiwari	*Mycobacterium abscessus*	Antiseptic	Infection outbreak
2003	Gajadhar	*Pseudomonas*	Antiseptic	Survey
2005	Ebner	*Burkholderia cepacia*	Disinfectant	Pseudo-outbreak
2005	Fisher	*Pseudomonas aeruginosa*	Antiseptic	Infection outbreak
2006	Lo Cascio	*Burkholderia cenocepacia*	Disinfectant	Infection outbreak
2007	Siebor	*Pseudomonas fluorescens; Achromobacter xylosoxidans*	Disinfectant	Pseudo-outbreak
2008	Lee CS	*Burkholderia cepacia*	Antiseptic	Infection outbreak
2010	Hakuno	*Pseudomonas fluorescens;Burkholderia cepacia; Aeromonas* species	Antiseptic	No infections
2014	Kampf	*Achromobacter* species; *Serratia marcescens*	Disinfectant	Survey
2015	Kupfahl	*Achromobacter* species	Disinfectant	Survey
2015	Hugon	*Achromobacter denitificans*	Disinfectant	Infection outbreak; contaminated surfaces
2016	Tandel	*Burkholderia cepacia*	Antiseptic	Pseudo-outbreak
2021	FDA	*Burkholderia cepacia complex* *Ralstonia pickettii*	Hand sanitizer	No reported infections
2022	Boyce	*Serratia marcescens; Achromobacter xylosoxidans*	Disinfectant	No infections; contaminated high-touch surfaces

* Centers for Disease Control and Prevention

** Originally classified as *Pseudomonas cepacia (mulivorans)*

Episodes of contamination involved skin antiseptics (24), surface disinfectants (17), a product used as both antiseptic and disinfectant (1), and a hand sanitizer (1). The most common QAC-resistant microorganisms responsible for contaminated disinfectants and antiseptics were *Pseudomonas* species (17), *Burkholderia* species (13), *Achromobacter* species (8), and *Serratia marcescens* (7) (Table 3). The types of infections reported in 29 episodes of contamination included bloodstream infections (15 episodes), wound infections (5), skin abscesses (2), septic arthritis (2), meningitis (2), urinary tract (3), ear cartilage infections (1), respiratory tract (1), and IV catheters (1), with more than one type of infection reported in several episodes [91, 92]. Contamination of surfaces in patient rooms occurred in three episodes [25, 113]. In one of the three episodes, bloodstream infections may have resulted from contamination of intravascular catheters by aerosols created by spraying of the implicated disinfectant, or by contamination of catheter materials from contaminated surfaces [113]. Contamination of surfaces in healthcare environments has also led to healthcare personnel hand contamination [96].

The frequency of reported episodes of contaminated disinfectants is probably an underestimate of the actual frequency. Kampf et al. [111] surveyed disinfectant buckets in 15 German hospitals and found that 42% of disinfectant buckets (often containing QAC-based products) from 11 hospitals were contaminated with *Achromobacter* spp. or *Serratia.* Kupfahl et al. [112] reported that 47% of 30 buckets containing QAC disinfectants in four medical centers were contaminated with up to 1 × 10^4^ CFU of *Achromobacter* spp. During the time period when buckets were sampled, an *Achromobacter* strain with the same pulsed-field gel electrophoresis (PFGE) pattern as that recovered from several contaminated disinfectant buckets was responsible for a nosocomial infection. The direction of spread (buckets to patient vs. patient to buckets) was not established.

The mechanisms responsible for the QAC resistance of the contaminating pathogens were not determined in most episodes, due to the lack of appropriate molecular methods or laboratory resources when contamination was discovered. In one study, resistance was attributed to decreased adsorption of BAC to the cell surface and changes consistent with an efflux pump [24]. *S. marcescens* isolates recovered from contaminated footbaths in several dairy farms were not killed when inoculated into a fresh in-use concentration of BAC, which reduced *S*. *marcescens* ATCC 13,880 by > 5 log_10_ [118]. The study suggested that reduced susceptibility was not due to decreased membrane permeability, but may have been due in part to the ability to form biofilm. In a recent study, whole genome sequencing revealed that *S. marcescens* recovered from an in-use hospital disinfectant contained *sdeXY*, *sdeAB*, *smfY*, and a *sugE*-like gene, which have been shown in earlier studies to encode for efflux pumps that confer reduced susceptibility to QAC compounds [25, 118]. The potential role of other mechanisms of resistance including biofilm formation was not assessed.

### Combined tolerance or resistance to QACs and antibiotics

Publications dealing with combined resistance or tolerance to both QACs and antibiotics have often used the terms cross-resistance or co-resistance. Cross-resistance is due to one mechanism that results in reduced susceptibility to two or more dissimilar agents. Examples include a single multidrug efflux pump that exports both QACs and antibiotics, or changes in cell membrane structure that affect both types of compounds [119, 120]. Co-resistance occurs when dissimilar mechanisms of reduced susceptibility are linked by two or more resistance genes that encode for unrelated resistance mechanisms. Examples would be linkage of genes encoding for QAC efflux pumps and genes encoding antibiotic resistance by production of an inactivating enzyme or by an altered intracellular target [27, 52, 121]. However, it is important to note that articles referring to cross-resistance and co-resistance often describe pathogens with QAC tolerance combined with antibiotic resistance.

### Combined QAC and antibiotic resistance in nonlaboratory settings

Several investigators who obtained pathogens from community settings or clinical isolates from hospitals have described isolates with tolerance (reduced susceptibility) to QACs plus clinically-defined antibiotic resistance [22, 40, 52, 89, 122, 123]. A 2008 study found a significant correlation between BAC tolerance and clinically-defined antibiotic resistance among Gram-negative bacteria found in home settings [124]. A number of these studies did not establish if the combined QAC tolerance and antibiotic resistance was due to cross-resistance or co-resistance. Instances of combined resistance to QACs and antibiotics have occasionally resulted in clinically significant consequences. For example, QAC resistance combined with antibiotic resistance (based on currently existing Clinical Laboratory Standards Institute breakpoints) has occurred in bacteria recovered in several episodes of contamination of in-use antiseptics or disinfectants [25, 103, 104, 113, 125–127]. In one episode caused by *S. marcescens*, co-resistance was due at least in part to the presence of multiple QAC efflux pumps and antibiotic resistance that was most likely due to an inducible chromosomally-mediated AmpC beta-lactamase, which is common in *S. marcescens* [25, 128]. In several of the above-mentioned episodes, contaminating pathogens with combined resistance to QACs and antibiotics caused healthcare-associated infections [103, 113, 125, 126].

Hospital sink drains contaminated with pathogens with high BAC or DDAC MICs and clinically-defined antibiotic resistance is another example of how combined resistance to QACs and antibiotics may be of clinical significance. For example, in one hospital, multidrug resistant extended-spectrum beta-lactamase producing *Enterobacter cloacae* strains with elevated BAC and DDAC MICs (64–512 ug/ml) recovered from hospital sink drain biofilms had the same PFGE type as strains responsible for healthcare-associated infections among patients cared for on the ward with contaminated sinks [129]. When the practice of pouring a DDAC-based disinfectant down sink drains was replaced by using a bleach-based solution and biofilms were removed from sinks, the incidence of infections decreased shortly thereafter, suggesting that the sinks may have been the source of healthcare-associated infections [129]. Sink-related outbreaks of infection are likely due at least in part to droplet transmission of pathogens from sinks to the hands of healthcare personnel, with subsequent spread to patients [130].

### Potential role of QAC use on antibiotic resistance

It has been stated that there is no evidence that use of antiseptics or disinfectants selects for antibiotic-resistant microorganisms in nature, or that such mutants survive in nature [34, 131]. Nonetheless, the widespread use of QAC disinfectants and growing frequency of multidrug-resistant healthcare-associated pathogens has increased concerns about whether or not use of QAC-based disinfectants promotes the emergence of tolerance or resistance to antibiotics, or to combined resistance to both antibiotics and QACs. Evidence cited in support of this concern includes the in vitro adaptive studies mentioned above wherein repeated or prolonged exposure to QACs resulted in increases in QAC and antibiotic MICs, sometimes to levels equal to in-use disinfectant concentrations and clinically relevant increases in antibiotic MICs [5, 78, 81–84].

For example, an adaptive study found that exposing *Ps. aeruginosa* to BAC resulted in both increased BAC MICs and mutations in the polymixin resistance gene *pmrB* and increased tolerance to polymixin [27]. A recent adaptive study repeatedly exposed *E. coli* to BAC in liquid media in a manner that was designed to mimic repeated application of disinfectant on surfaces yielded BAC-tolerant presistent mutants that had no change in BAC MIC, but had 2-fold increases in MICs to ampicillin or ciprofloxacin, or both [31]. Although the persistent cells had a slower growth rate, they had improved ability to survive sub-inhibitory concentrations of antibiotics, suggesting that the BAC-tolerant persistent mutants may have a selective advantage in environments where antibiotics are present [31]. It may be useful to conduct similar experiments on hard surfaces using commerical disinfectants to see if similar mutant persisters also occur in real-world settings.

The occurrence of mobile genetic determinants (e.g. plasmids, transposons, integrons) that carry genes that encode for tolerance to QACs and others that encode for tolerance to antibiotics also raises the concern that QAC use might lead to decreased susceptibility to antibiotics [52, 55, 132]. A study that utilized an *E. coli* donor strain carrying an RP4 plasmid carrying resistance to ampicillin, kanamycin and tetracycline evaluated the effect of exposure to dodecyl dimethyl benzyl ammonium chloride (DDBAC) on plasmid conjugation [132]. Exposure to DDBAC promoted conjugative transfer of the antibiotic-resistance plasmid to a recipient *E. coli* strain.

Buffet-Bataillon et al. [52] identified clinical strains of *E. coli* that contained a class 1 integron containing genes encoding for reduced susceptibility to trimethoprim-sulfamethoxazole and QACs (*dfrA/Sul1* and *qacE∆1*, respectively). The same strains also showed overexpression of the efflux pump element *tolC* and its regulators, which was associated with reduced susceptibility to QACs and ciprofloxacin [52]. The presence of both qacH and a gene (*sul3*) encoding increased tolerance to sulfomamides on a conjugative plasmid in *Proteus mirabilis* raises the possibility of co-transfer of QAC and antibiotic tolerance [55]. *E. coli* strains recovered from retail meats in China have been shown to contain plasmids with integrons that carry genes encoding for tolerance to both QACs and antibiotics [133]. The authors of the latter study demonstrated that the plasmids could be horizontally transferred to an *E. coli* recipient strain. While the occurrence of genes that confer tolerance or resistance to both QACs and antibiotics (co-resistance) raises the possibility that widespread use of QACs may promote antibiotic resistance, dissemination of clinically-significant antibiotic resistance by this mechanism has not been established in non-laboratory settings.

It seems likely that emergence of QAC-resistant pathogens in contaminated in-use disinfectants occurs when bacteria with intrinsic mechanisms of QAC resistance and ability to form biofilms (e.g., *Pseudomonas* spp. and *Burkholderia cepacia*) are repeatedly exposed to sublethal QAC concentrations due to suboptimal disinfection practices. It is not clear if exposure to the QAC selects for clinically-relevant levels of antibiotic resistance similar to what has been shown in *in vitro studies*, or if the pathogens were already antibiotic-resistant when inadvertently introduced into disinfectant containers.

In vitro studies suggest that sink drain biofilms may provide a milieu in which repeated or prolonged exposure of mixed communities of bacteria to QACs may result in enrichment of Gram-negative organisms (e.g., *Pseudomonas* spp.) with intrinsic tolerance to QACs, the ability to encode for efflux pumps and form biofilm, and perhaps to degrade QACs, resulting in sub-inhibitory concentrations of QACs that allow the bacteria to survive and develop resistance to both QACs and antibiotics [21, 27, 37, 39, 77, 83]. The same may be true for *Burkholderia* spp., which also possess many of the same microbial properties [38, 70, 134]. Alternatively, exposure of pathogens to QACs in this setting may result solely in emergence of QAC tolerance or resistance in pathogens which were already antibiotic-resistant when they became trapped in sink drain biofilm.

Currently, there is no evidence that accumulation on hard surfaces of sub-inhibitory concentrations of QACs has resulted in selection of antibiotic-resistant pathogens. In fact, there are very little published data regarding QAC levels on surfaces routinely disinfected with QAC-based products. A recent study found that a single application to surfaces previously exposed to a QAC disinfectant resulted in only minor increases in the QAC concentrations on school desks [135]. Repeated application of BAC to surfaces in buses led to increasing concentrations that peaked after one to two weeks [136]. A one-time sampling of surfaces in a hospital nursing station revealed BAC levels ranging from 6.9 to 76.6 ug/100 cm [2, 10].

### Research gaps

Although QAC-based disinfectants and antiseptics have been widely used for decades, there are issues related to their use that are poorly understood and require additional research. A brief list of issues that would benefit from additional research is noted below:

- Our understanding of QAC tolerance and resistance would benefit from greater utilization of standardized definitions of these terms by investigators.
- Development and adoption of standard protocols for exposing pathogens to in-use concentrations of products may yield better estimates of bacterial resistance to biocides [87].
- Longitudinal studies of the level of QAC accumulation on surfaces repeatedly disinfected with QAC-containing disinfectants and the pathogens recovered from such surfaces may provide data regarding whether or not sub-inhibitory QAC concentrations on surfaces may promote QAC resistance or antibiotic resistance, as noted in some laboratory-based adaptive experiments.
- Data are needed regarding the tolerance and resistance of newer QAC compounds, such as bis- and tris-QACs and polymeric QACs, where other moieties such as organosilanes, an oxazoline homopolymer, or silver are attached to mono- or bis-QACs [137–139].
- Additional field studies are needed to establish the frequency with which in-use solutions of dilutable QAC-based disinfectants are contaminated.
- Pathogens with combined tolerance/resistance to QACs plus antibiotic resistance that have been recovered from contaminated in-use disinfectants should be examined using methods that can establish the molecular mechanisms for combined resistance to determine the likelihood that exposure to QACs co-selected for or induced antibiotic resistance, or if antibiotic resistance was due solely to the presence of preexisting mechanisms of resistance resulting from widespread antibiotic use.
- The potential role that sink drain biofilms may contribute to the spread of antibiotic-resistant pathogens requires further studies [129]. Longitudinal studies of sink drains utilizing whole genome sequencing and metagenomic methods similar to those conducted by Johnson RC et al. [140] and Constantinides B et al. [141] might yield information regarding possible co-selection or induction of antibiotic resistance by QACs if the studies include identification of resistance mechanisms of both QACs and antibiotics. Ideally, such studies should include measurement of QAC levels in biofilm and QAC MICs/MBCs of pathogens imbedded in biofilm, given the fact that one in vitro study found that exposure of a sink biofilm microcosm to very low levels of BAC or disinfectant solution did not result in detection of significantly increased QAC or antibiotic resistance levels [88].

## Discussion

QAC-containing disinfectants are widely used due to a number of factors, including their activity against many bacteria, fungi and enveloped viruses, low cost (especially in concentrated forms), relatively good materials compatibility, and much less offensive odor when compared to sodium hypochorite (bleach) or peracetic acid-containing products. However, spore-forming organisms and mycobacteria are intrinsically resistant to QACs, and Gram-negative bacteria are generally less susceptible to QACs than Gram-positive bacteria. In vitro studies involving exposure of pathogens to increasing concentrations of QACs have shown that bacteria can utilize a variety of mechanisms to develop tolerance, or less commonly, resistance to QACs and/or antibiotics. To date, there are relatively limited examples of *de novo* development of QAC tolerance or resistance in real-world settings. Compared to other disinfectants and antiseptics, those containing QACs as the sole active agent are more likely to become contaminated with Gram-negative bacteria. Multiple episodes of contamination of in-use antiseptic and disinfectant solutions by pathogens with resistance to QACs (sometimes combined with antibiotic resistance) have resulted in clusters or outbreaks of healthcare-associated infections. Combined resistance to both QACs and antibiotics has also been reported in pathogens imbedded in sink biofilms exposed to a QAC disinfectant, with possible transmission of pathogens to patients.

To avoid contamination of in-use disinfectants, re-usable buckets in which dilutable QAC-based disinfectants are used should be thoroughly cleaned and dried before new disinfectant is added [25, 41]. It is important to test the in-use concentrations of dilutable QAC disinfectants following manual dilution, and after new containers of concentrated product are attached to automated dilution systems. Daily testing the concentration of dilutable QAC solutions in buckets should be considered if cotton or microfiber cloths are used, especially if multiple wipes are allowed to soak in buckets [117]. Exposure of pathogens to low and ineffective concentrations may also occur by failing to remove organic material (including biofilms), adsorption of QAC to the wipe material, or application of products at a suboptimal pH [29, 69, 117, 142]. QAC-based disinfectants should not be used to wipe the tops of blood culture bottles prior to injecting blood into culture bottles [105]. Because multiple outbreaks related to contaminated BAC-based antiseptics have occurred, the Centers for Disease Control and Prevention Guideline for Disinfection and Sterilization in Healthcare no longer lists QAC-based antiseptics as one of the recommended uses of QACs in healthcare settings [34]. Of note, contaminated disinfectant/antiseptic products have been based on either BAC, DDAC, BAC + DDAC, or cetrimide-chorhexidine gluconate [25, 101, 104, 108, 113, 114, 125, 126, 143]. Disinfectants containing one or more QACs plus additional active agents (e.g., ≥ 15% alcohol) have not been reported to be contaminated.

## Conclusions

The increased use of QAC-containing disinfectants during the COVID-19 pandemic has heightened concerns regarding the possible impact that widespread QAC use and environmental build-up may have on promoting antimicrobial resistance among pathogens in healthcare and other settings. However, despite some evidence from laboratory-based studies, currently there is a lack of conclusive evidence that QAC use has contributed to widespread emergence of antimicrobial resistance in real-world settings. Additional studies of the evolution of pathogens with combined QAC tolerance/resistance and antibiotic resistance in real-world settings and of the mechanisms that may be responsible for combined resistance are needed to better understand the possible role that QAC use may play in promoting clinically-significant antibiotic resistance.

## Data Availability

Data used to form tables and figures are available in articles available in the PubMed database.
